# Supplementary material for: Anti-Cancer Potential of Linear β-(1→6)-D-Glucan from Agaricus bisporus on Estrogen Receptor-Positive (ER+) Breast Cancer Cells
Source: Molecules. 2024 Oct 9;29(19):4781. doi: 10.3390/molecules29194781 (PMC11482474; doi:10.3390/molecules29194781)

Figure S1: Relative expression of BCL2 gene. MCF-7 cells were treated with BDG16 at 100, 300, 500 and 1000 µg/mL for 48 h. Statistical analyses were performed by one-way analysis of variance (ANOVA) followed by Bonferroni's post-test, selected pairs. No significant differences were observed on cells treated with BDG16 in comparison with control.

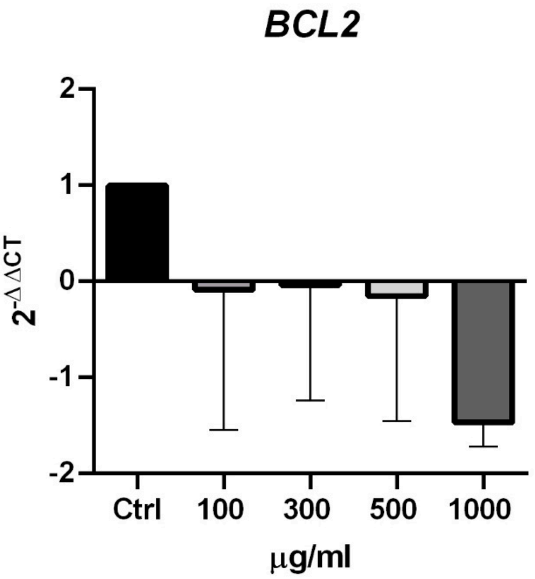

Supplement: Supplementary file 1 [file molecules-29-04781-s001.zip › molecules-3233253-supplementary.pdf]
